# Utilizing GO/PEDOT:PSS/PtNPs-enhanced high-stability microelectrode arrays for investigating epilepsy-induced striatal electrophysiology alterations

**DOI:** 10.3389/fbioe.2024.1376151

**Published:** 2024-03-25

**Authors:** Meiqi Han, Yu Wang, Luyi Jing, Gucheng Yang, Yaoyao Liu, Fan Mo, Zhaojie Xu, Jinping Luo, Qianli Jia, Yuxin Zhu, Hanwen Cao, Xinxia Cai, Juntao Liu

**Affiliations:** ^1^ State Key Laboratory of Transducer Technology, Aerospace Information Research Institute, Chinese Academy of Sciences, Beijing, China; ^2^ School of Electronic, Electrical and Communication Engineering, University of Chinese Academy of Sciences, Beijing, China

**Keywords:** microelectrode arrays, temporal lobe epilepsy, striatum, graphene oxide, PEDOT:PSS

## Abstract

The striatum plays a crucial role in studying epilepsy, as it is involved in seizure generation and modulation of brain activity. To explore the complex interplay between the striatum and epilepsy, we engineered advanced microelectrode arrays (MEAs) specifically designed for precise monitoring of striatal electrophysiological activities in rats. These observations were made during and following seizure induction, particularly three and 7 days post-initial modeling. The modification of graphene oxide (GO)/poly (3,4-ethylenedioxythiophene):polystyrene sulfonate (PEDOT:PSS)/platinu-m nanoparticles (PtNPs) demonstrated a marked reduction in impedance (10.5 ± 1.1 kΩ), and maintained exceptional stability, with impedance levels remaining consistently low (23 kΩ) even 14 days post-implantation. As seizure intensity escalated, we observed a corresponding increase in neuronal firing rates and local field potential power, with a notable shift towards higher frequency peaks and augmented inter-channel correlation. Significantly, during the grand mal seizures, theta and alpha bands became the dominant frequencies in the local field potential. Compared to the normal group, the spike firing rates on day 3 and 7 post-modeling were significantly higher, accompanied by a decreased firing interval. Power in both delta and theta bands exhibited an increasing trend, correlating with the duration of epilepsy. These findings offer valuable insights into the dynamic processes of striatal neural activity during the initial and latent phases of temporal lobe epilepsy and contribute to our understanding of the neural mechanisms underpinning epilepsy.

## 1 Introduction

Epilepsy, affecting approximately 65 million individuals globally, is a common neurological condition characterized by recurrent, uncontrolled seizures. These seizures arise from abnormal, synchronized neuronal firing in the brain (Stafstrom and Carmant, 2015; Devinsky et al., 2018). Temporal lobe epilepsy (TLE), the most common type of intractable epilepsy (Bernhardt et al., 2019), typically originates in specific brain regions, especially the hippocampus (Cendes, 2005), and spreads through various neural networks. The interconnected nature of cortical and subcortical regions means activity in one area can influence others, leading to extensive seizure activity (Englot et al., 2015; Paz and Huguenard, 2015; Duncan et al., 2016). The interconnected nature of cortical and subcortical regions means activity in one area can influence others, leading to extensive seizure activity. The significant impact of epilepsy on individuals’ health, daily life, and professional activities poses a major healthcare challenge, emphasizing the need for in-depth research into the neural circuits and cellular mechanisms driving this disorder (Nabbout and Kuchenbuch, 2020).

The basal ganglia network, which plays a crucial role in coordinating motor functions and cognitive processes (DeLong and Wichmann, 2010), integrates diverse inputs from the neocortex, thalamus, and hippocampus (Alexander and Crutcher, 1990). Although it does not directly initiate epileptic seizures, the network is instrumental in modulating the propagation and regulation of both focal and generalized epileptic seizures (Antoine and Moshé, 2002; Deransart and Depaulis, 2002; Goldstein, 2008). Within this network, the striatum, through dopamine and GABAergic transmissions, significantly influences the onset and dissemination of epilepsy (Turski et al., 1991). The role of dopamine in epileptic seizures is hypothesized to be governed by the distinct activation patterns of D1 and D2 dopamine receptors. Striatal D2 receptor activation can play a protective role in pilocarpine-induced epilepsy (Turski et al., 1987; Al-Tajir and Starr, 1991). The specific neural information changes and underlying mechanisms in the striatum during chronic epilepsy remain elusive, necessitating the utilization of high-spatio-temporal resolution tools for their detection and analysis.

The advent of micro-electromechanical systems (MEMS) technology has greatly enhanced the development and application of microelectrode arrays (MEAs). These arrays are increasingly used to detect electrophysiological and electrochemical signals in the brain, offering deeper insights into brain function and propelling neurological research forward (Johnson et al., 2008; Xiao et al., 2019). Characterized by their multi-channel, high-resolution capabilities, these arrays can effectively capture electrical signals from specific brain regions post-implantation (Xu et al., 2022). Platinum nanoparticles (PtNPs), known for their antioxidant properties and surface characteristics, have found widespread use in electronics and biology (Jan et al., 2021). The electrodes are co-electrochemically modified with graphene oxide and poly (3,4-ethylenedioxythiophene):polystyrene sulfonate (PEDOT:PSS), embedding graphene within PEDOT:PSS to reduce impedance and enhance biocompatibility (Ko et al., 2021).

In rodent studies, models such as electrical kindling, maximal shock seizure, lithium-pilocarpine, and kainic acid are frequently used to induce TLE (Pinel and Rovner, 1978). To examine the changes in neural activity within the striatum as a component of the circuit involved in TLE, we utilized specially designed MEAs to capture action potential (spike) and local field potential (LFP) recordings in rats with lithium-pilocarpine-induced epilepsy. MEMS technology was used to fabricate MEAs for recording striatal electrophysiological signals in the normal control group (Control), seizure, three days (3 days), and seven days (7 days) after modeling. Our analysis revealed the formation of hypersynchronous abnormal discharges in the striatum during seizures, accompanied by changes in action potentials and field potentials post-seizure induction.

In summary, our study successfully fabricated 30-channel MEAs capable of real-time, high-resolution *in vivo* recording of electrophysiological signals in the striatum of rats, both before and after epileptic seizures. This research aims to investigate and explore alterations in striatal neural signals during epileptic seizures and the early stages following epilepsy modeling from an electrophysiological perspective, providing novel insights into the role of the striatum in epileptic seizures and propagation circuits.

## 2 Materials and methods

### 2.1 Reagents and apparatus

Phosphate-buffered saline (PBS) was acquired from Beijing Solarbio Science & Technology Co., Ltd. (China). The Chloral hydrate was procured from Aladdin (China). Graphene oxide (GO) was sourced from XFNANO (China). Poly (sodium 4-styrenesulfonate) (PSS) was sourced from Shanghai HEROCHEM Co., Ltd. (China). The 3,4-Ethylenedioxythiophene (EDOT) was procured from Sigma-Aldrich (United States). The 1,1′-Dioctadecyl-3,3,3′,3′-tetramethylindocarbocyanine perchlorate (Dil) and chloroplatinic acid (H_2_PtCl_6_) were purchased from Acmec Biochemical Co., Ltd. (Shanghai, China). The plumbous acetate trihydrate [Pb(CH_3_COO)_2_·3H_2_O] was purchased from Haohong Scientific Co., Ltd. (Shanghai, China). The isoflurane anesthesia machine, accompanying isoflurane anesthetic, and anesthetic gas recovery tank were acquired from RWD Life Sciences Co., Ltd. (Shenzhen, China). High-purity chemicals (Macklin Biochemical Corporation, Shanghai, China) such as anhydrous Lithium chloride (LiCl, 99%), scopolamine methyl bromide (98%), and pilocarpine hydrochloride (99%).

The stereotaxic frame (Stoelting, United States) and micropositioner (David KOPF instrument, United States) were used for position determination and electrode implantation. The electrochemical workstation (Gamry Reference 600, United States) was used for site modification and electrochemical characterization. Brain slices were precisely prepared using a clinical cryostat from Leica Biosystems (United States). For examination and analysis of MEAs and brain sections, we used two microscopes, one from Olympus Corporation (Japan) and the other purchased from Leica Biosystems (United States).

### 2.2 Design and fabrication of the MEAs

**FIGURE 1:**
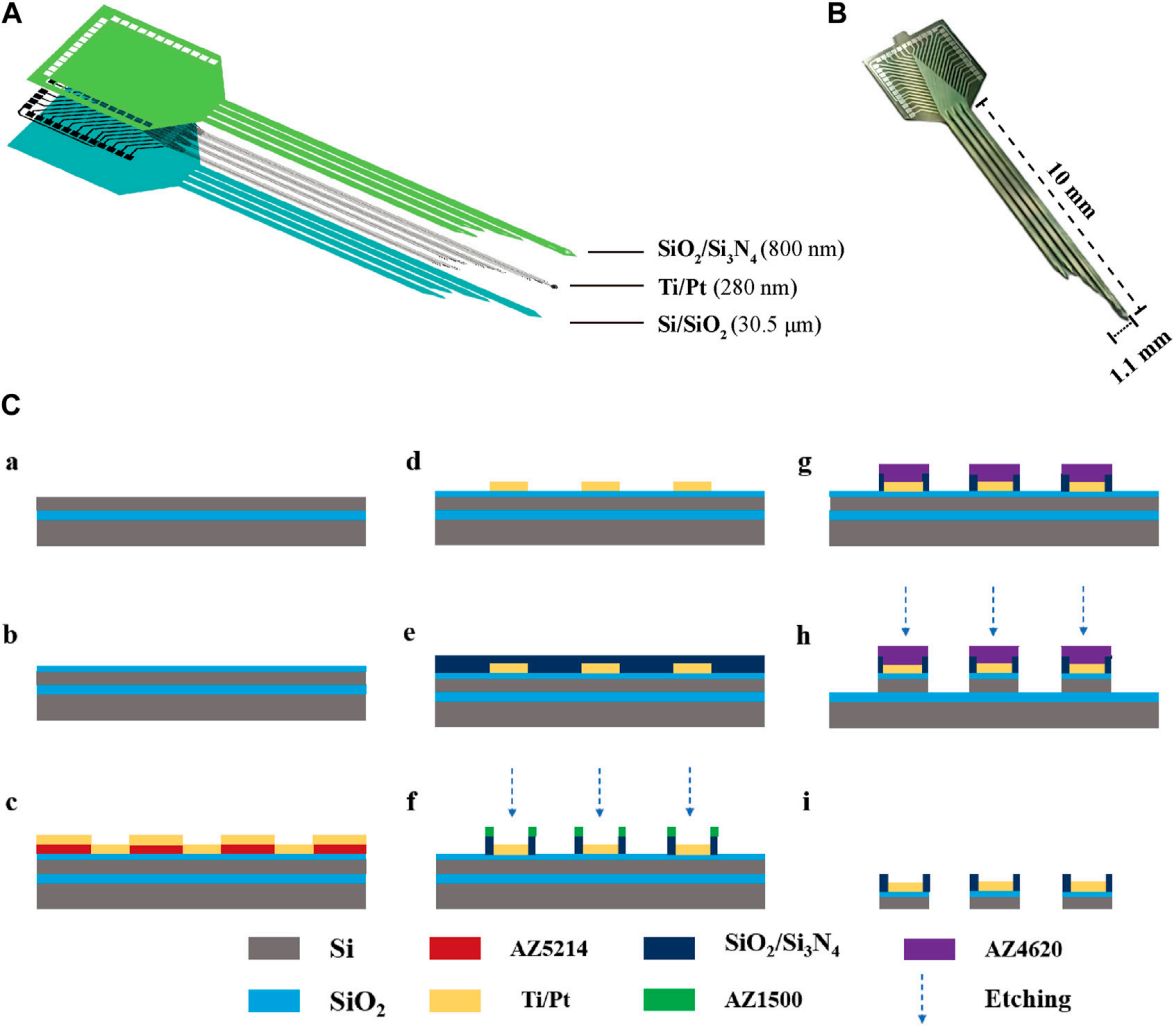
The design and fabrication of MEAs. **(A)** Decomposition diagram of the electrode structure, consisting of a Si base layer, a SiO_2_ insulating layer, a Ti/Pt metal layer, and a SiO_2_/Si_3_N_4_ insulating layer. **(B)** MEAs **(C)** Flow chart of electrode fabrication: (a) SOI sheet. (b) Thermal oxidation of silicon in the top layer of SOI generates silica. (c) Lithography (AZ5214) generates a pattern of metal sputtering and sputters Ti/Pt. (d) The sputtered metal layer is patterned using the lift-off process. (e) Inductively coupled plasma chemical vapour deposition deposited SiO_2_/Si_3_N_4_. (f) Lithography (AZ1500) generates site and pad photoresist patterns and performs insulating layer (SiO_2_/Si_3_N_4_) etching. (g) Lithography (AZ4620) generates a photoresist pattern of the MEA shape. (h) The SiO_2_/Si layer underwent an etching process. (i) MEAs were released by KOH wet etching after sealing.

The MEAs were fabricated using MEMS, as previously reported. Figure 1A illustrates the structure of the MEAs, which employ silicon on insulator (SOI) as the primary material. This includes a Si base layer (30 μm), a SiO_2_ insulating layer (500 nm), a Ti/Pt metal layer (30 nm/250 nm), a SiO_2_/Si_3_N_4_ insulating layer (300 nm/500 nm), and a GO/PEDOT:PSS/PtNPs nanocomposite. The physical drawing of MEAs is depicted in Figure 1B, featuring 30 detection sites distributed across four electrode handles. The fabrication process for MEAs is presented in Figure 1C.

### 2.3 Nanomodification of the MEAs

The bare electrode is known to exhibit high impedance and suboptimal biocompatibility, which can impede the effective acquisition of electrophysiological signals. However, electrode sites that have been modified with GO/PEDOT:PSS/PtNPs nanocomposites demonstrate a substantial reduction in impedance, minimal phase delay, and enhanced electrical performance. Moreover, this modification enhances the stability of the electrode *in vivo*.

A platinum black plating solution was meticulously prepared by mixing H_2_PtCl_6_ (48 mM) and Pb (CH_3_COO)_2_ (4.2 mM) in a 1:1 volume ratio, followed by allowing the mixture to stand for 12 h before filtration. To prepare the GO/PEDOT:PSS suspension, 0.1 M PSS was initially integrated into the GO dispersion. This mixture was then sonicated for several hours to ensure complete mixing. Subsequently, we added EDOT (20 mM) to the above solution and then we sonicated it for 2 h.

The modification of the electrode sites with GO/PEDOT:PSS/PtNPs was executed using the Gamry 600 electrochemical workstation. Pt nanoparticles were grown on the site via chronoamperometry (−0.95 V, 60 s). The cyclic voltammetry technique was employed, utilizing a working electrode with a voltage scan range of 0–0.95 V and a scanning rate of 100 mV/s.

### 2.4 Animals

Male Sprague-Dawley rats weighing 250 g–300 g were utilized in this experiment. The rats were procured from Vital River Laboratory Animal Technology Co., Ltd. (Beijing, China). All rodents were individually housed in cages under a 12/12 h light-dark cycle. The ambient temperature was maintained at 25°C ± 2°C with a relative humidity of 50%–70%. Food and water were provided *ad libitum*. The animal experimentation protocols were conducted in strict adherence to ethical guidelines.

### 2.5 Experimental surgery

During the surgical procedure, rats were anesthetized with isoflurane (3%–5% for induction and 0.8%–1.5% for maintenance) and securely positioned on a stereotaxic apparatus. To prepare for craniotomy, the hair and scalp of the rats were carefully shaved using a razor, revealing the skull. The craniotomy site was accurately determined using specific coordinates: 1.32 mm anterior to the midline and 2.5 mm lateral (AP: 1.32 mm, ML: 2.5 mm), ensuring precise access to the targeted brain region. Five sites on the skull were selected using skull nails as force points for ground electrodes and dental cement, and a skull drill was used to form windows at target locations on the skull. Next, the MEAs were implanted into the brain area through an open-out window (DV: 7.5 mm). A wire was utilized to establish a connection between the shielded metal box, serving as ground, and the skull nail. Following the accurate placement of the electrode into the targeted position, it, along with the skull, was securely fixed using dental cement to ensure stability and precision during signal acquisition.

### 2.6 Pilocarpine-induced temporal lobe epilepsy in rats

The lithium-pilocarpine model, widely recognized internationally as a drug-induced rat model of TLE resembling the clinical condition is well-established (Reddy and Kuruba, 2013; Xie et al., 2023). In this model, activation of acetylcholine muscarinic receptors induces persistent generalized clonus, leading to seizures. First, LiCl (127 mg/kg) was injected intraperitoneally to sensitize the rats to pilocarpine. After 18–20 h, the rats received an intraperitoneal injection of scopolamine methyl bromide at a dosage of 1 mg/kg. This was administered to mitigate peripheral response damage during seizure episodes. After a waiting period of 30 min, an intraperitoneal injection of pilocarpine at 20 mg/kg was administered to induce seizures. In cases where the initial pilocarpine injection failed to trigger a grand mal seizure, additional doses of pilocarpine (12 mg/kg) were administered at 30-min intervals until a seizure reaching or exceeding level 4 on the Racine scale was observed (Racine, 1975). The establishment of successful epileptic models was determined by the occurrence of seizures that were at least level 4 on the Racine scale and lasted 1 h. One hour after the grand mal seizure, the seizures were terminated by intraperitoneal injection of 10% chloral hydrate (3 mL/kg).

### 2.7 Experimental design

**FIGURE 2:**
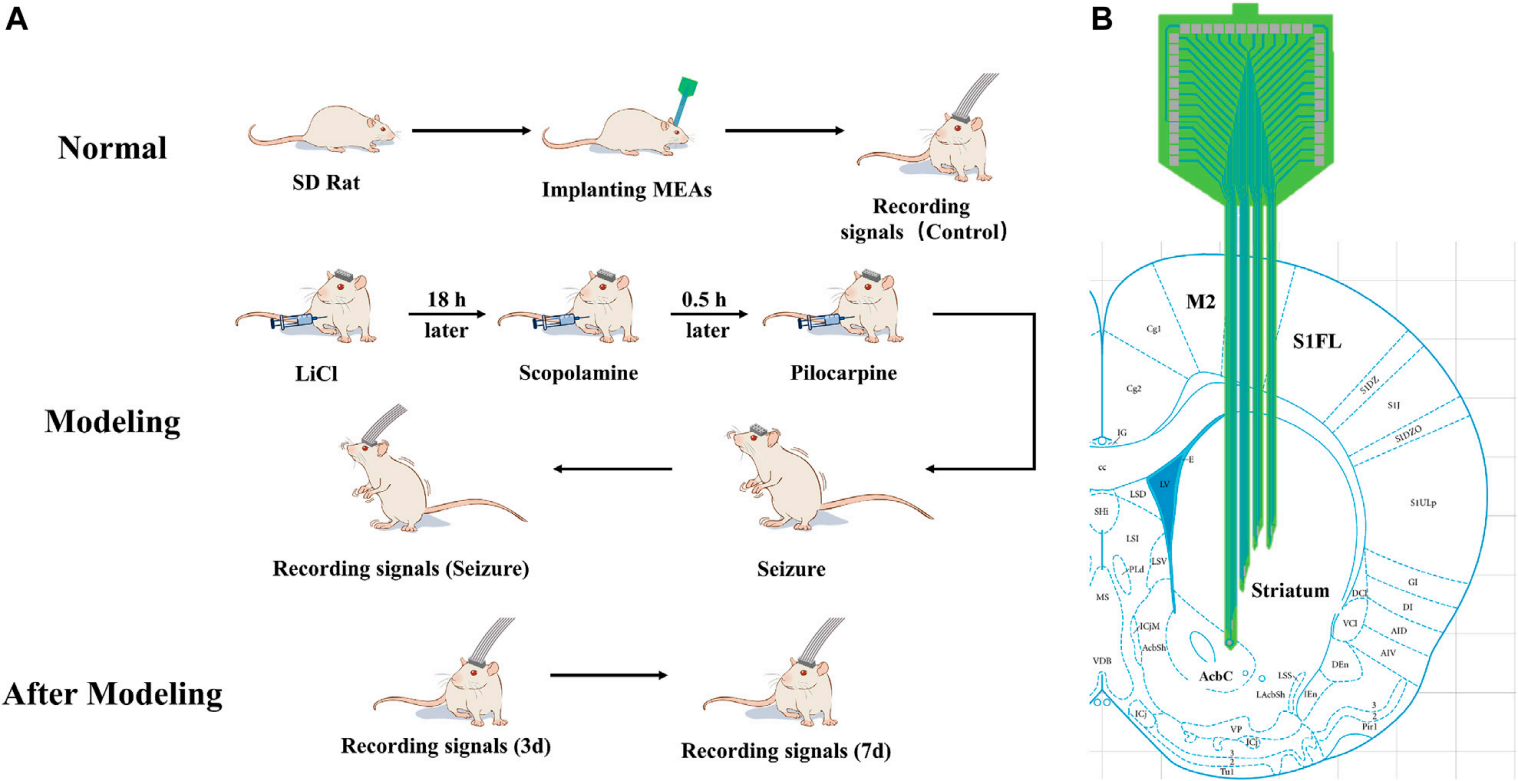
Schematic illustration of the experimental design **(A)** Scheme of animal experiments, including microelectrode implantation, epilepsy modeling, and signals recording. **(B)** Schematic representation of the implanted brain area.

The experimental design is shown in Figure 2A. The electrodes were first implanted into the normal rats striatum (Figure 2B). After implantation, electrophysiological signals were recorded from the normal rats striatal neurons and designated as the control group (Control) in our study. After 2–3 days, seizures were induced by pilocarpine, and electrophysiological signals were recorded. The rats were returned to the cage, and electrophysiological signals were detected on day 3 (3 days) and 7 (7 days) after modeling. Finally, the various stages of the induced seizures were compared and analyzed, and the data of the normal control group and three and 7 days after the induced seizures were compared and analyzed to obtain reliable conclusions.

### 2.8 Data recording and analysis

The electrophysiological signals from the striatum were recorded using a high-throughput neural signal acquisition device developed by our research group (AIRCAS-128, China). This instrument operates at a sampling rate of 30 kHz (He et al., 2021). LFPs were isolated using a low-pass filter with a range of 0–250 Hz, while spikes were extracted via a high-pass filter set above 250 Hz, applying a threshold set at thrice the baseline noise level.

For the classification and subsequent statistical analysis of spikes and LFPs, we employed NeuroExplorer 4 (Nex Technologies, United States) and Offlinesorter (Plexon, United States). The data were analyzed using the mean ± SD format. Statistical significance was determined by *p* < 0.05 (ANOVA test).

## 3 Results and discussion

### 3.1 Electrochemical characterization of GO/PEDOT:PSS/PtNPs -modified MEAs

**FIGURE 3:**
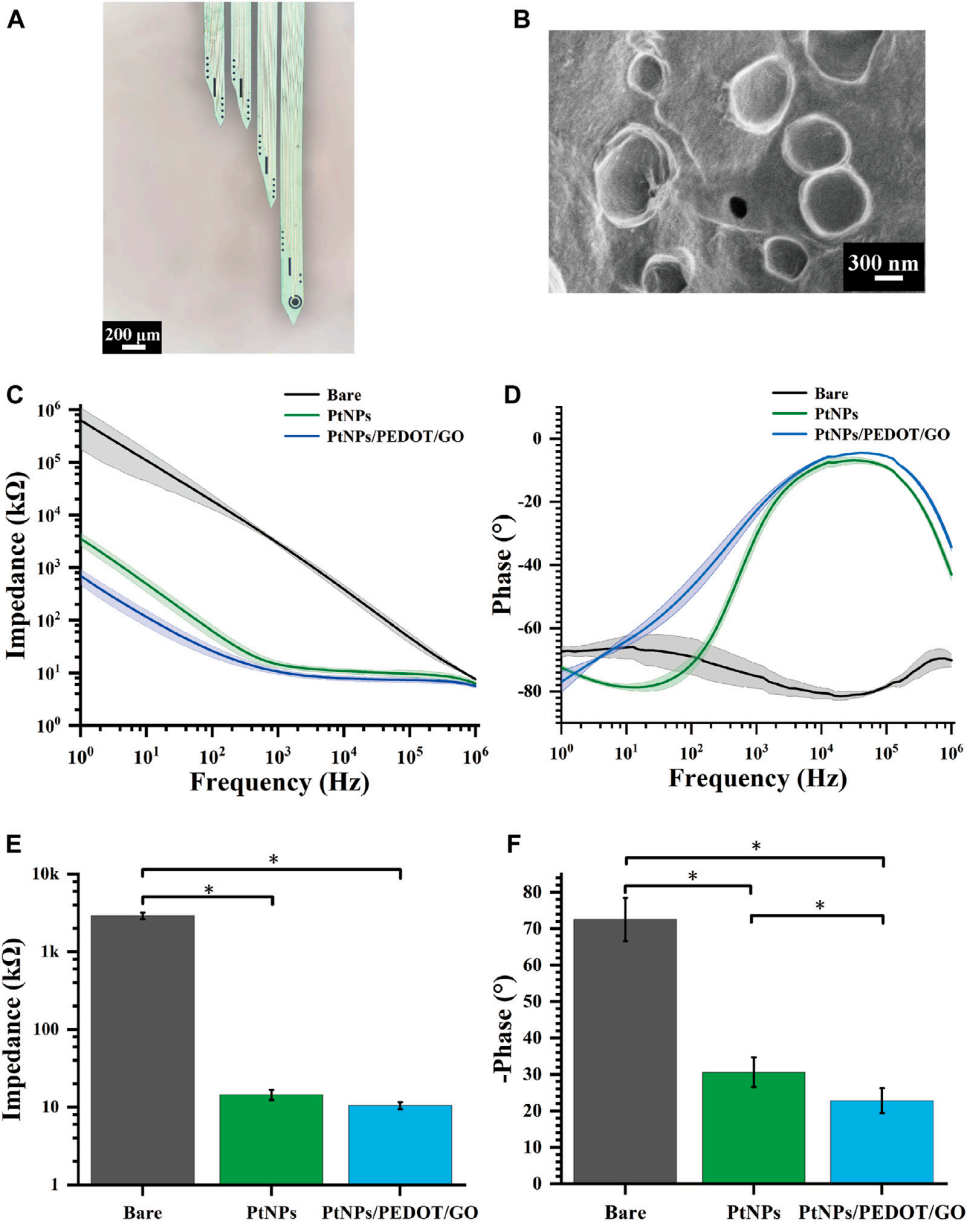
Electrochemical characterization of GO/PEDOT:PSS/PtNPs modified MEAs. **(A)** Microscopic view of MEAs modified by GO/PEDOT:PSS/PtNPs. **(B)** Scanning electron microscopy of GO/PEDOT:PSS/PtNPs modified electrode sites. **(C)** Impedance measurements across the frequency range of 1 Hz–10^6^ Hz. **(D)** Phase measurements across the frequency range of 1 Hz–10^6^ Hz. **(E)** Average impedance measurements at 1 kHz (*n* = 10,*p < 0.05). **(F)** Average phase measurements at 1 kHz (*n* = 10,*p < 0.05).

The MEAs, following detailed design, encapsulation, and modification processes, were successfully implanted in rats. Figure 3A illustrates a microscopic view of the GO/PEDOT:PSS/PtNPs-modified MEAs. Scanning electron microscopy (SEM) showed that the site surface after platinum black modification was rough and granular (Supplementary Figure S2A), while the GO/PEDOT:PSS-modified sites exhibited a smooth and stratified structure (Figure 3B). This alteration enhances the specific surface area, which is advantageous for improving electron transfer efficiency. Elemental composition analysis of the modified sites (Supplementary Figures S2B, D) indicated that compared to PtNPs modification, GO/PEDOT:PSS modification led to an increase in carbon, oxygen, and sulfur content at the electrode site. The weight percentage of elemental carbon increased from 12.34% to 48.64%, the weight percentage of elemental oxygen rose from 1.46% to 25.35%, and the weight percentage of elemental sulfur went from 0% to 12.7%. This change signifies successful material modification at the electrode sites.

During our investigation, the properties of the GO/PEDOT:PSS/PtNPs-modified MEAs were evaluated using electrochemical impedance spectroscopy in a PBS buffer solution. Across a frequency range spanning from 1 Hz to 10^6^ Hz, both the impedance and phase at the modified sites exhibited a significant reduction compared to the unmodified sites, as depicted in Figures 3C, D. This observation confirmed that the GO/PEDOT:PSS/PtNPs modification resulted in lowered impedance and phase delay.

A critical frequency often employed in neuroscience research is 1 kHz (Prasad and Sanchez, 2012). At this frequency, the impedance of the modified MEAs decreased markedly from 2907 ± 289 kΩ (bare) to 15 ± 2.13 kΩ (PtNPs), and then to 10.5 ± 1.1 kΩ (GO/PEDOT:PSS/PtNPs) (Figure 3E), and the phase delay reduced from 72.5° ± 5.9° (bare) to 30.6° ± 4.1° (PtNPs), and then to 22.79° ± 3.38° (GO/PEDOT:PSS/PtNPs) (Figure 3F). The results demonstrate that the GO/PEDOT:PSS/PtNPs modification significantly enhances the electrical performance of the sites.

We used cyclic voltammetry experiments to verify the stability of the compound modification. After cyclic voltammetry (voltage range −0.6–0.8 V, number of cycles 230) (Supplementary Figure S3A), the average impedance of the site increased from 11.77 kΩ to 13.39 kΩ (an increase of 14%). The mean phase delay increased from 13.5° to 14.8° (an increase of 9.6%) (Supplementary Figure S3B). The small change in average impedance and phase indicated that the modified electrode sites had a high stability.

Additionally, impedance testing of sites after 14 days of implantation in rats revealed that the *in vivo* environment led to an increase in average impedance from 10.5 kΩ to 23 kΩ. However, this value remained below 50 kΩ, as shown in Supplementary Figure S4, indicating a relatively minor decline in electrode performance throughout the experiment.

### 3.2 Striatal neurons fire abnormally in synchrony during pilocarpine-induced seizures

The electrode channels’ names and their approximate locations within the implanted brain regions are illustrated in Supplementary Figure S5. Our electrophysiological recording system captures signals that include spikes and the LFP, extracted from the original signal. LFP represents a collection of electrophysiological activities from numerous neurons surrounding sites, reflecting relatively macroscopic neural activity. Power spectral density (PSD) can provide insights into energy distribution across different frequencies.

To enhance the analysis of distinct neural information alterations in the striatum during epileptic seizures, we meticulously categorized pilocarpine-induced epileptic seizures into four sequential stages: quiet period, preictal period, middle period, and grand mal period. The “quiet period” was designated as the 10–15 min interval immediately after the injection of pilocarpine. The “preictal period” was identified by the presence of high amplitude oscillations in the local field potentials (LFPs), as observed in the signal acquisition window. The “middle period” was defined as occurring 20 min subsequent to the preictal phase. Lastly, the “grand mal period” was established as the phase beginning 20 min after the middle period, characterized by more pronounced seizure activities.

**FIGURE 4:**
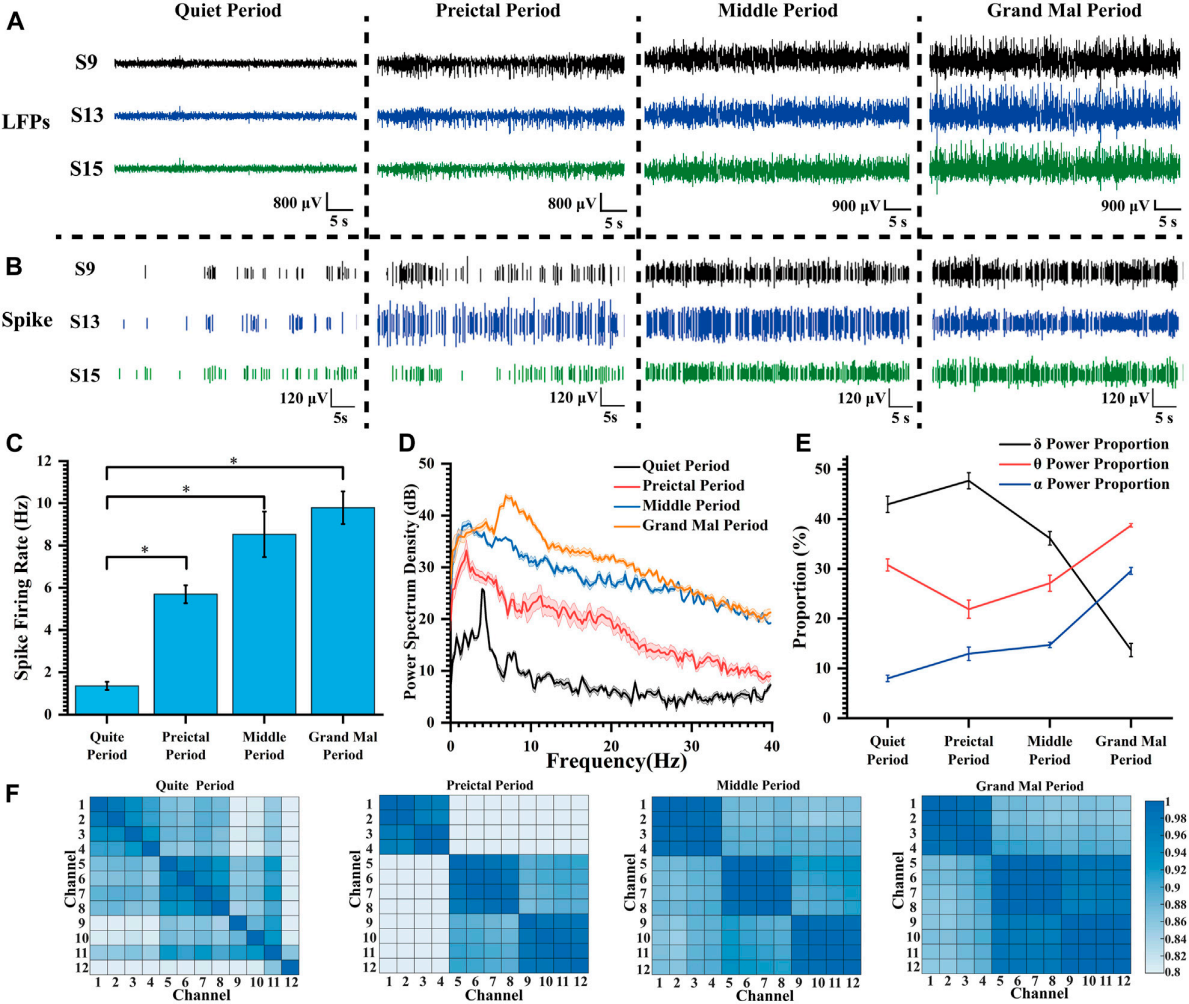
Characteristics of the electrophysiological signals during pilocarpine-induced seizures. **(A)** LFP changes during pilocarpine-induced seizures. **(B)** Spike changes during pilocarpine-induced seizures. **(C)** Spike firing rate at different degrees of seizure induction (*p < 0.05). **(D)** Power spectral density in LFPs during different stages of seizure induction. **(E)** The proportion of LFP power during different stages of seizure induction. **(F)** Heat map of the correlation matrix across various channels during distinct stages of induced seizure activity.

We selected three channels with representatives to show the LFP and spike signals during the quiet period and at various degrees of seizure (Figures 4A, B). As the epilepsy induction deepened, the density of spike discharges in rats increased to seven times the original density. Furthermore, the amplitude of LFP also increased significantly approximately 10-fold, resulting in high-amplitude and high-frequency signals. These findings indicate that the striatum is affected and exhibits severe abnormal discharge during seizure induction.

Following the widely accepted conventions in electroencephalogram signal analysis, the electrophysiological frequency bands are categorized as follows: the delta (δ) band spans from 0 to 4 Hz, the theta (θ) band spans 4–8 Hz, the alpha (α) band extends from 8 to 13 Hz, and the beta (β) band covers frequencies between 13 and 30 Hz. As illustrated in Figure 4C, with increasing severity of epilepsy, there was a progressive increase in mean spike firing rate from 1.36 Hz to 9.79 Hz, indicating a gradual intensification of abnormal epileptic discharges. In Figure 4D, the peak power of the quiet period appeared at about 4 Hz; the peak power frequency shifted to lower frequencies during the preictal period, but the amplitude did increase rapidly, and the power amplitude continued to rise during the middle period. During the grand mal period, the power peak moved rapidly to high frequency, and the power also increased further.

In our thorough analysis, we illustrated the percentage distribution of various frequency bands during seizure episodes in a line chart (Figure 4E). Our observations revealed a progressive increase in the proportion of the δ frequency range, accompanied by a concurrent decrease in the θ frequency range and an elevation in the α frequency range at epilepsy onset. With escalating seizure severity, there was a significant reduction in the proportion of the delta band during the middle and grand mal stages. Conversely, there was a substantial increase in the proportion of the theta frequency band, emerging as the predominant frequency. Additionally, we observed an elevation in activity within the α frequency band. This θ-α activity closely correlates with regions implicated in seizure propagation during epilepsy (Sip et al., 2021). We propose that as seizure severity increases, slow waves initially manifest within striatal regions and progressively evolve into high-frequency oscillations that delineate seizure progression.

In the field of statistics, the Pearson correlation coefficient, which ranges from −1 to 1, serves as a metric for assessing the correlation between two variables. A higher absolute value indicates a stronger correlation (negative values indicate negative correlations). In our study, 12 channels at varying depths (chS1-S12) were chosen, with chS1-chS4 close to the ventral striatum and other channels gradually implanted at lower depths and located in the dorsolateral striatum. The correlation between different channels in each stage of seizure induction was explored and presented in the form of heat maps (Figure 4F). We considered the four adjacent channels as a group, with chS1-chS4 as group 1, chS5-chS8 as group 2, and chS5-chS8 as group 3. It can be found that there was no regular correlation between channels in the quiet period, and the correlation of channels within the group increased during the preictal period, and the correlation of channels between group 2 and group 3 increased. With the deepening of seizure depth, the correlation between each channel and all channels in the group increased continuously, and the correlation between group 2 and group 3 increased significantly. According to the change of correlation, it can be speculated that the neurons begin to discharge abnormally synchronously when the seizures occur, and the abnormal discharge of neurons in the striatum becomes more and more synchronized with the seizures, which can verify that the epilepsy disease is a hypersynchronous abnormal discharge phenomenon. This fits with the relevant definition that epilepsy is abnormally enhanced neuronal synchrony and related research results (Fisher et al., 2005; San-Juan and Rodríguez-Méndez, 2023). Relevant studies can be carried out according to related phenomena to explore the specific neural mechanism of epileptic seizures.

### 3.3 Chronic changes of electrophysiological signals of striatum before and after modeling

**FIGURE 5:**
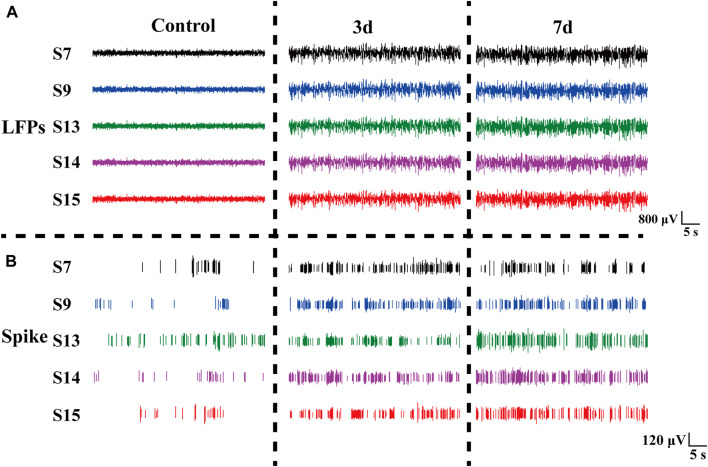
Electrophysiological signals at different days in rats. **(A)** LFP changes in normal Control rats (Control), three days (3 days), and seven days (7 days) after modeling. **(B)** Spike changes in normal control rats (Control), three days (3 days), and seven days (7 days) after modeling.

Rats induced with pilocarpine exhibited signs of fatigue, heightened sensitivity to auditory, visual, and other stimuli, and experienced weight loss. As demonstrated in Figure 5, we meticulously selected the original electrophysiological signals from five representative channels. Notably, the spike discharge density in the striatum of rats exhibited a significant increase on day 3 and 7 post-modeling, in comparison to the normal control group. The spike discharge density increased approximately 1.5-fold. Concurrently, there was an emergence of epileptiform discharges in the field potential, indicating altered neuronal activity.

### 3.4 Enhanced spike firing rates and reduced intervals in neuronal activity

**FIGURE 6:**
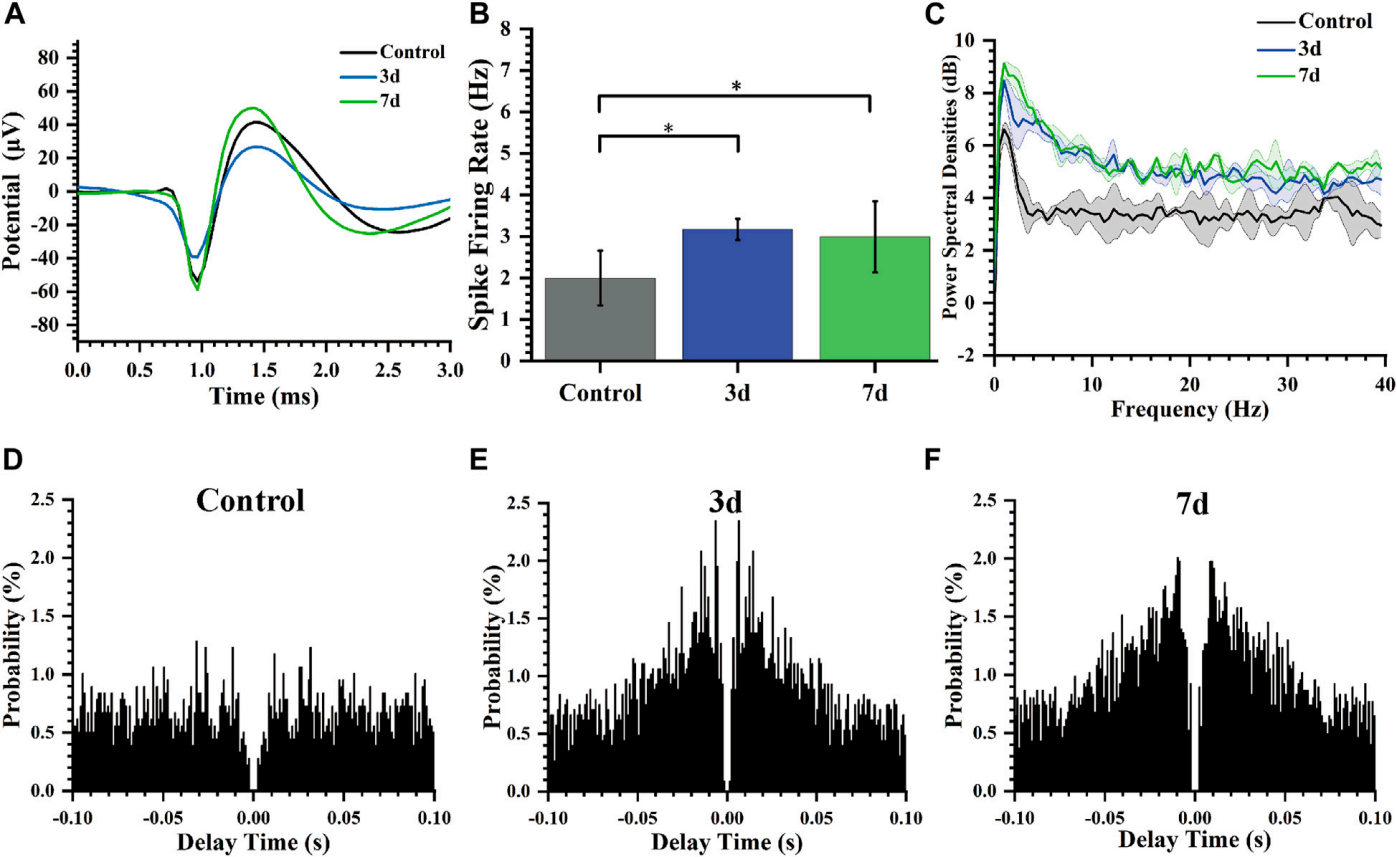
Characteristics of spikes of rats at different days. **(A)** Average Spike waveform of rats in different stages. **(B)** Spike firing rate of rats in different days (*p < 0.05). **(C)** Spike power spectral density of rats in different days. **(D)** Autocorrelogram of the normal control group. **(E)** Autocorrelogram of experimental group 3 days after modeling. **(F)** Autocorrelogram of experimental group 7 days after modeling.

In our study, we performed statistical analysis on the spike firing rate of rats at normal, 3 days, and 7 days after modeling. Figure 6A shows the spike average waveform in these three stages, and the shape of the waveform is unchanged, but the amplitude is different. Our analysis of the distinct segments revealed a statistically significant variation in the spike firing rate of striatal neurons (Figure 6B). Specifically, there was an increase in firing rate from 1.99 ± 0.66 Hz to 3.16 ± 0.25 Hz, and then to 2.99 ± 0.85 Hz on day 3 and 7 post-modeling respectively. Furthermore, we conducted a comprehensive calculation and analysis of the average power spectral density of spikes across different periods which demonstrated an increased peak power in rats’ spike activity following the modeling process (Figure 6C).

Autocorrelogram showed the conditional probability (*y*-axis) of a spike at delay-time (*x*-axis) on the condition that there was a spike at time zero. The spike autocorrelation analysis for representative channels was also performed and presented as histograms (Figures 6D–F). Compared to the control group, the model group (3 days, 7 days) exhibited a higher magnitude in autocorrelation peak along with a reduced latency period for this peak occurrence time point indicating notable modifications in electrophysiological activity of striatal neurons including decreased firing delay time and increased firing rate after epilepsy modeling.

### 3.5 Revealing a gradual augmentation in LFP power over time

**FIGURE 7:**
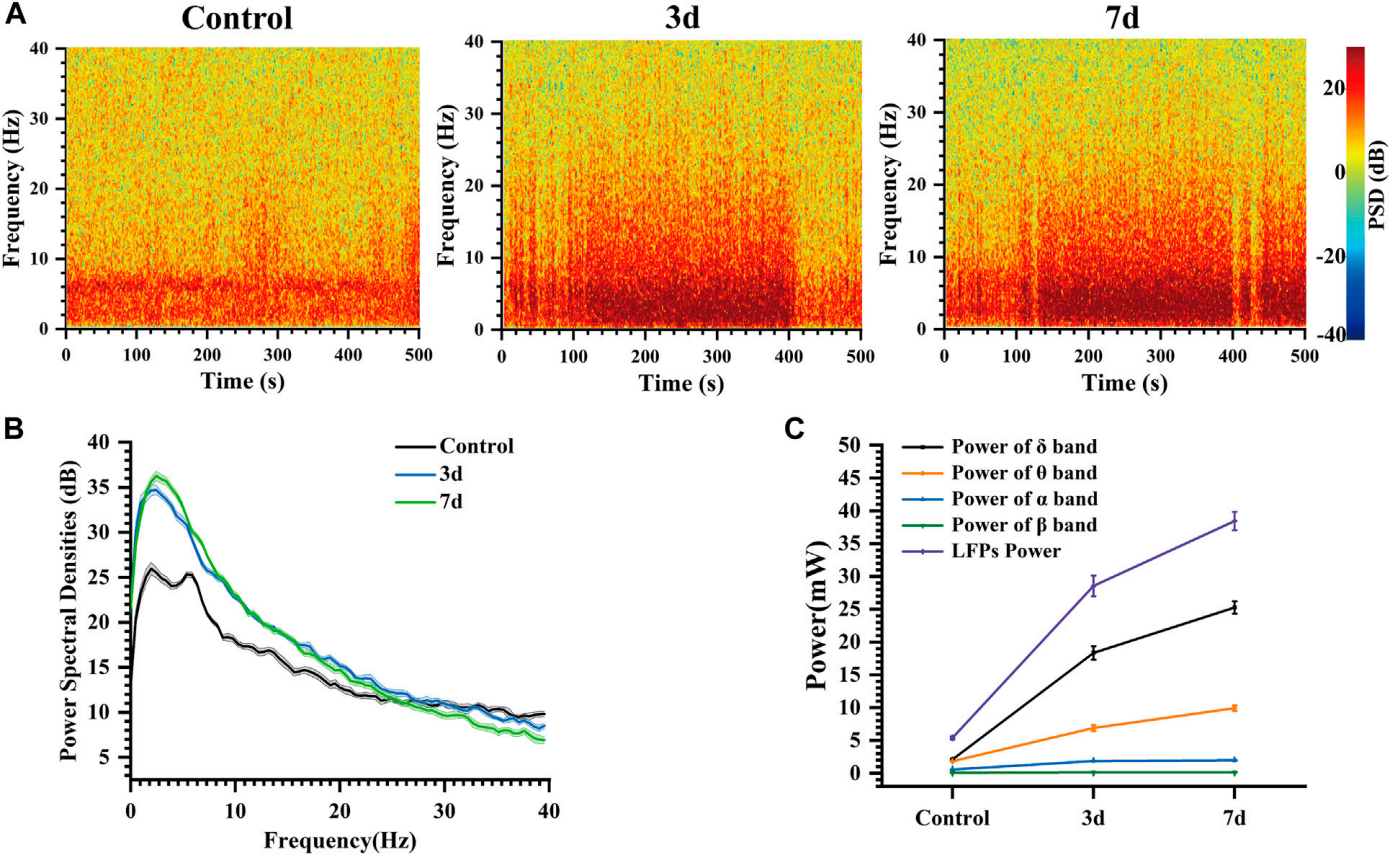
Characteristics of the LFP of rats at different days. **(A)** Spectrogram of LFPs at different days. **(B)** LFP power spectral density at different days. **(C)** Power of each frequency band and total power of representative channels at different days.

The color in the power spectral density heatmap represented the magnitude of the power spectral density, with the redder color representing the higher power at this point. In Figure 7A, compared to normal rats, the power spectral density heat map of rats after three and seven days of epilepsy modeling exhibited a significant increase in power at frequencies ranging from 0 to 10 Hz. Additionally, the simultaneous presence of multiple high-frequency energy was observed, indicating the manifestation of epileptiform electrical activity. In our study, Figure 7B illustrates the LFP power spectral density across different stages within the frequency range of 0–40 Hz. During the Control stage, power predominantly concentrated in the δ and θ bands; however, both power amplitude and peak increased during the 3 days and 7 days stages. The peak of power spectral density increased from 26 dB (Control) to 34.7 dB (3 days), and then to 36.26 dB (7 days). Notably, energy remained primarily concentrated in δ frequencies which aligns with previously reported findings (Gambardella et al., 1995; Yang et al., 2012; Freund et al., 2022).

At the same time, the changes in the power of each frequency band and the total power of LFP in these three different days were analyzed (Figure 7C). Our findings indicate that LFP total power increased over time following modeling, with the primary contribution to this increase coming from δ and θ bands, while α and β bands exhibited less change. Mean power in the δ band increased from 2.09 mW (Control) to 18.35 mW (3 days), and then to 25.27 mW (7 days). Mean power in the θ band increased from 1.82 mW (Control) to 6.89 mW (3 days), and then to 9.93 mW (7 days). These results suggest that abnormal discharges become increasingly intense during this process, leading to a progressively stronger impact of epilepsy on the striatum. As time progressed, LFP total power continued to rise due primarily to increases in δ and θ band activity within the striatum indicating an elevated risk for seizure associated with more frequent abnormal discharges (Pelliccia et al., 2013; De Stefano et al., 2022).

This demonstrates that the impact of epilepsy on the striatum persists over time, resulting in abnormal discharge phenomena that indicate early changes in neural information within the striatum following epilepsy modeling. These findings are valuable for facilitating early screening and diagnosis of epilepsy.

## 4 Conclusion

In our study, we developed and fabricated specific MEAs to capture electrophysiological signals. Furthermore, we enhanced the electrode sites by electrochemically modifying them with GO/PEDOT:PSS/PtNPs. The modified sites exhibited reduced impedance and phase delay, excellent biocompatibility, and exceptional stability.

The electrophysiological signals recorded by the MEAs in our study provide compelling evidence of epilepsy’s profound impact on neuronal activity within the striatum. These electrophysiological data were analyzed during seizure induction, as well as before and after seizure modeling (latency). Our findings demonstrate that as seizure induction deepens, abnormal discharges may propagate throughout the entire striatum via theta-alpha band activity, giving rise to progressively more correlated hypersynchronous epileptic discharges. Compared to pre-modeling rats, spike firing rates increased and inter-firing intervals shortened post-modeling, resulting in frequent abnormal discharges. Additionally, high-frequency epileptiform discharges were observed in the LFP recordings along with an increase in peak power within the power spectral density. LFP power gradually escalated over time following modeling initiation, paralleling a progressive escalation of seizure risk and reflecting the evolution of epilepsy from its latent period to a chronic state.

MEAs were employed to collect and analyze electrophysiological data from the epileptic striatum, facilitating investigation into the dynamic processes underlying epilepsy. This study provides valuable research methods and tools for elucidating the mechanisms of epilepsy and studying its early onset. Specifically, our focus lies in conducting electrophysiological investigations during the initiation and early stages of experimentally induced temporal lobe epilepsy. However, further exploration is required to examine detection techniques during the chronic phase of chronic temporal lobe epilepsy in future studies.

## Data Availability

The raw data supporting the conclusion of this article will be made available by the authors, without undue reservation.
