# Supplementary material for: Utilizing GO/PEDOT:PSS/PtNPs-enhanced high-stability microelectrode arrays for investigating epilepsy-induced striatal electrophysiology alterations
Source: Front Bioeng Biotechnol. 2024 Mar 25;12:1376151. doi: 10.3389/fbioe.2024.1376151 (PMC11022210; doi:10.3389/fbioe.2024.1376151)
Supplement: Supplementary file 1 [file DataSheet1.PDF]

Supplementary Material

Utilizing GO/PEDOT:PSS/PtNPs-Enhanced High-Stability Microelectrod Arrays for Investigating Epilepsy-Induced Striatal Electrophysiology Alterations

Meiqi Han[1,2], Yu Wang[1,2], Luyi Jing[1,2], Gucheng Yang[1,2], Yaoyao Liu[1,2], Fan Mo[1,2], Zhaojie Xu[1,2], Jinping Luo[1,2], Qianli Jia[1,2], Yuxin Zhu[1,2], Hanwen Cao[1,2], Xinxia Cai[1,2*], Juntao Liu[1,2*]

[1] State Key Laboratory of Transducer Technology, Aerospace Information Research Institute, Chinese Academy of Sciences, Beijing, China

[2] School of Electronic, Electrical and Communication Engineering, University of Chinese Academy of Sciences, Beijing, China

*** Correspondence:** Juntao Liu: liujuntao@mail.ie.ac.cn, Xinxia Cai: xxcai@mail.ie.ac.cn

1 Supplementary Texts and Figures

1.1 Supplementary Texts

1.1.1 Histological verification of the implantation site

We conducted a verification process to confirm the placement of MEAs in the striatum using tissue sections. Before implantation, we applied fluorescent dye (Dil) onto the tips of the MEAs. This coating procedure was repeated twice every thirty minutes to ensure proper adherence of Dil dye onto the MEAs. Following the completion of implantation, we dehydrated the brain by administering normal saline (0.9%) and paraformaldehyde (4%) through an intra-cardiac injection. The brains were then extracted and sequentially immersed in sucrose solution (20% and 30%). These brain slices were carefully positioned on slides, allowing us to adjust our field of view for microscopic observation and image capture purposes (Supplementary Figure S1).

1.2 Supplementary Figures

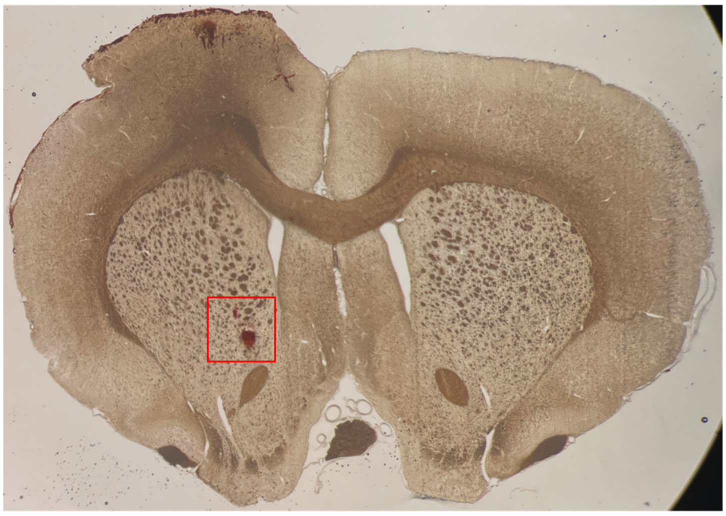

Figure S1. The implantation location of the MEA

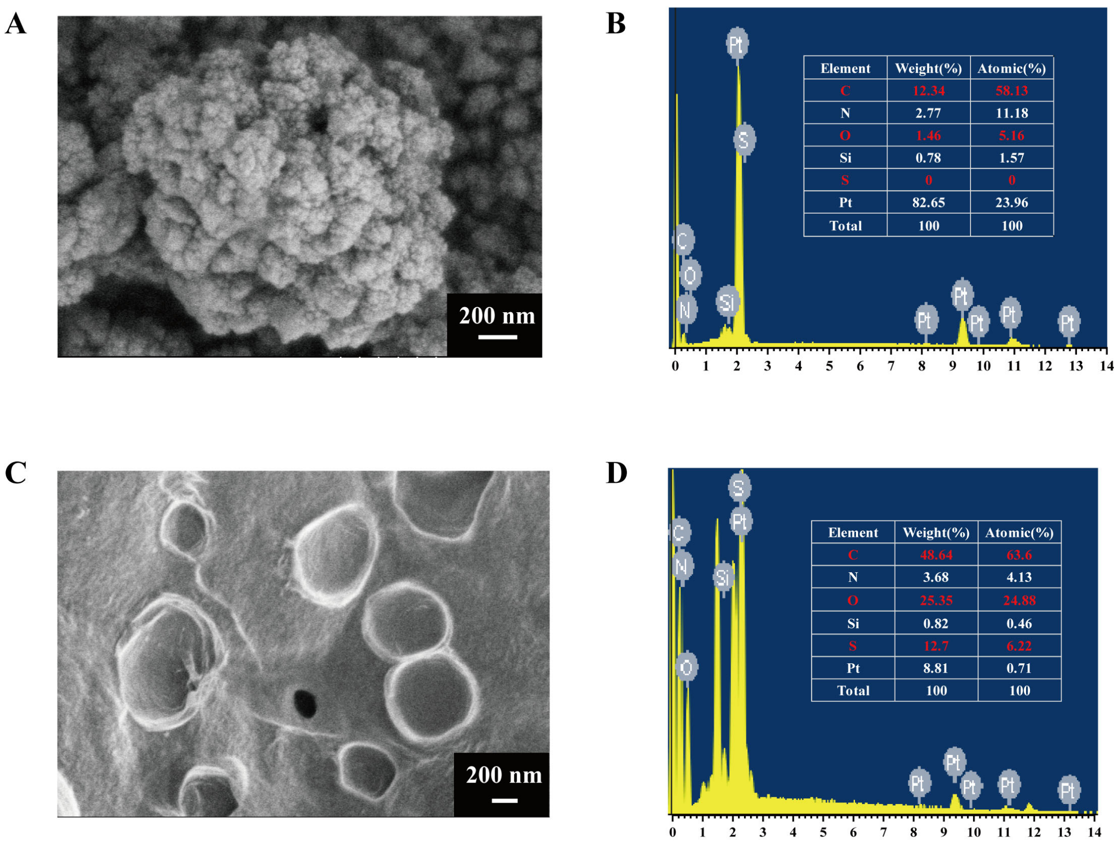

Figure S2. Scanning electron microscopy of modified electrode sites and elements. (A) Scanning electron microscopy of PtNPs modified electrode sites. (B) Composition of PtNPs modified electrode

sites. (C) Scanning electron microscopy of GO/PEDOT:PSS/PtNPs modified electrode sites. (D) Element composition of electrode sites modified by GO/PEDOT:PSS/PtNPs.

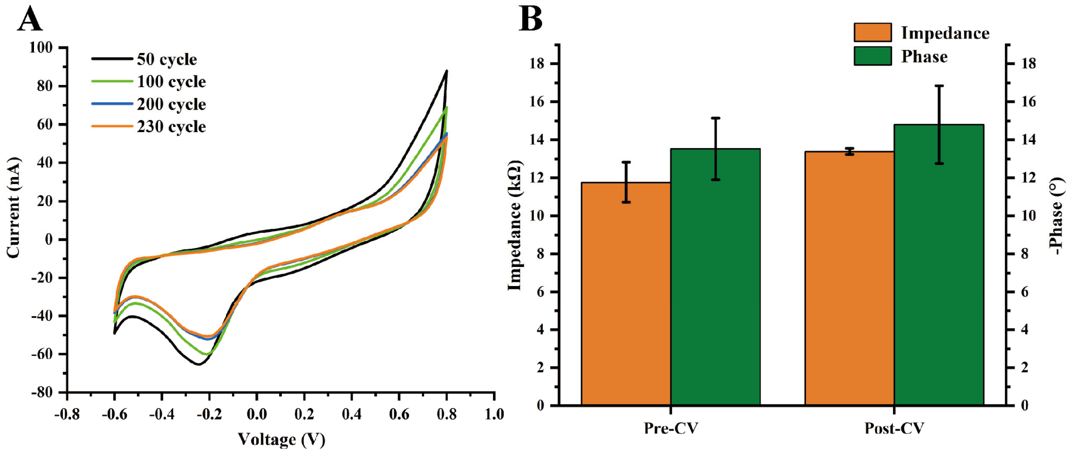

Figure S3. Cyclic voltammetry electrode stability test and sites performance characterization. (A) Voltage-current curves during cyclic voltammetry testing. (B) Impedance and phase representations of sites before and after cyclic voltammetry(230 cycles) at 1kHz.

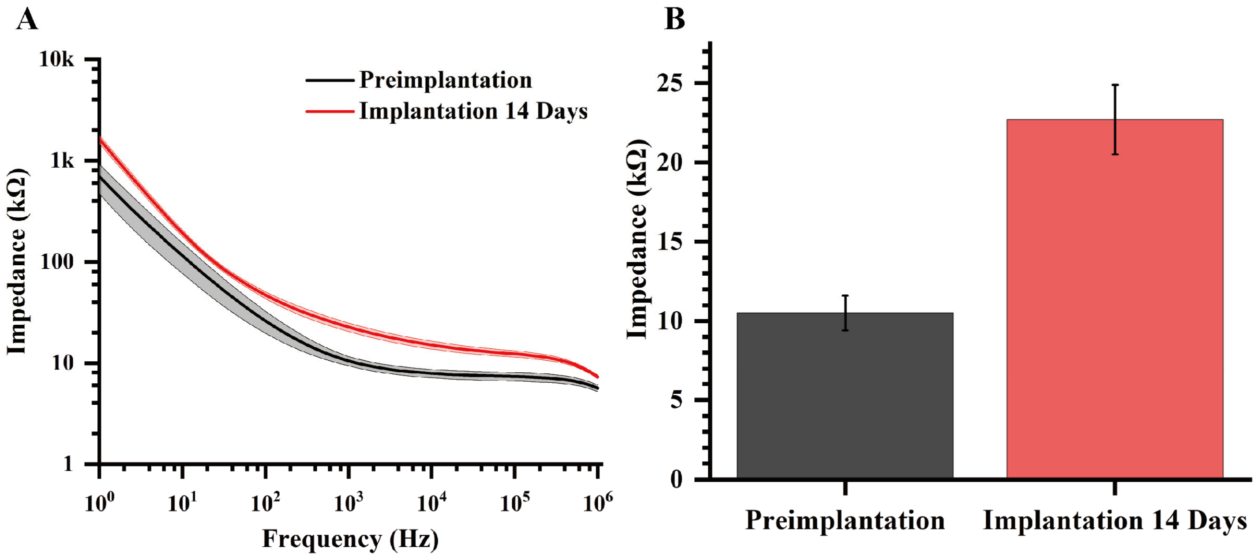

Figure S4. Electrode sites characteristics before implantation and fourteen days after implantation. (A) Impedance characterization before implantation and 14 days after implantation from 1 Hz to 10^6 Hz. (B) Average impedance characteristics before implantation and 14 days after implantation at 1kHz.

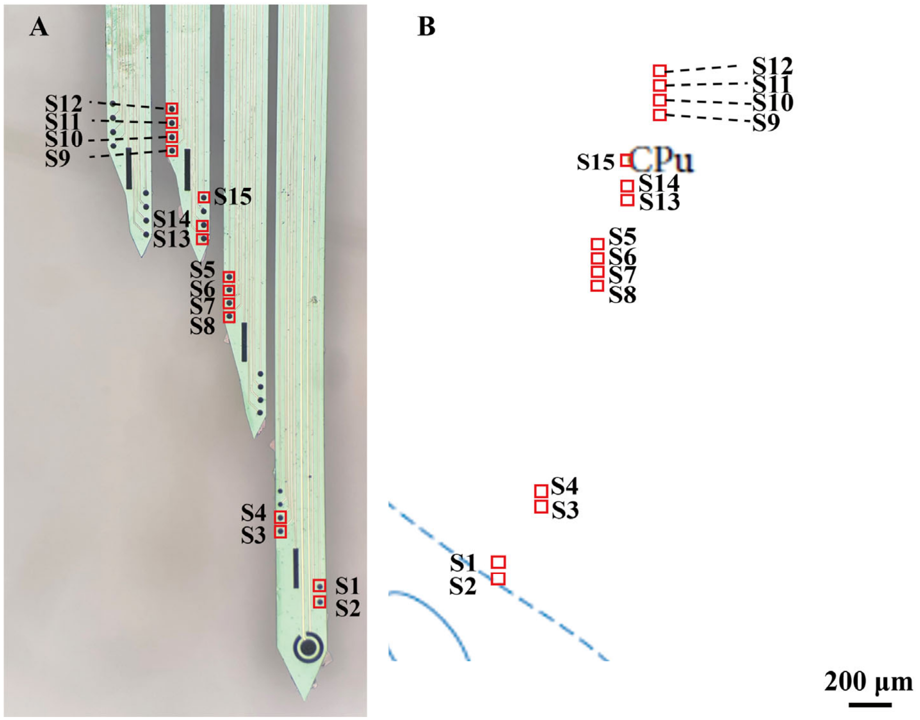

Figure S5. Distribution of electrode sites. (A) Electrodes on the physical distribution channel. (B) Schematic diagram of the corresponding brain regions of each channel after electrode implantation.
